# The R156H variation in IL-12Rβ1 is not a mutation

**DOI:** 10.1186/1824-7288-39-12

**Published:** 2013-02-14

**Authors:** Esther van de Vosse, Jaap T van Dissel, Loredana Palamaro, Giuliana Giardino, Francesca Santamaria, Rosa Romano, Anna Fusco, Silvia Montella, Mariacarolina Salerno, Matilde Valeria Ursini, Claudio Pignata

**Affiliations:** 1 Department of Infectious Diseases, Leiden University Medical Center, Albinusdreef 2, 2333 ZA, Leiden, The Netherlands; 2 Department of Translational Medical Sciences, “Federico II” University, Naples, Italy; 3 International Institute of Genetics and Biophysics, CNR, Naples, Italy

**Keywords:** *IL12RB1*, IL-12Rβ1, Immunodeficiency, Mutation, Mycobacterial disease

## Abstract

Palamaro *et al.* describe a child with recurrent bronchopneumonia and very high IgE levels in which a variation, R156H, was found in the *IL12RB1* gene that encodes the IL-12Rβ1 chain. Based on the absence of this variation in 50 unrelated individuals they conclude it is a mutation. We (van de Vosse and van Dissel) feel there is no reason to suspect a defect in IL-12 signaling based on the clinical data, nor evidence for a functional defect in IL-12 signaling in this patient. In addition, the variation is not novel and known as a polymorphism. Without any functional evidence that R156H is a mutation, the current claim is not substantiated.

Palamaro *et al.* respond to argue that the amino acid substitution, R156H described in the described case exerts a summatory effect, as a genetic cofactor, along with an additional and still unidentified molecular alteration of the same pathway.

## 

Letter to the Editor - Esther van de Vosse and Jaap T. van Dissel

We have read with interest the article by Loredana Palamaro *et al.*[1] about a child with recurrent bronchopneumonia and very high IgE levels in which a mutation was found in the *IL12RB1* gene that encodes the IL-12Rβ1 chain of both the IL-12 and IL-23 receptor.

In individuals with IL-12Rβ1 deficiency, or other deficiencies leading to familial atypical mycobacteriosis (OMIM 209950), typically infections with non-tuberculous mycobacteria (NTM), and in case of IL-12Rβ1 deficient patients also non-typhoid salmonellae, are observed. These infections are often recurrent, difficult to treat, and/or disseminated throughout the body [2]. Very seldom infections confined to lung parenchyma are found, and in those cases mostly *Mycobacterium tuberculosis* is involved, a pathogen with a strong preference for the lung. Conversely, in individuals with NTM lung tissue infections, no defects in the IL-12/IFN-γ pathway are identified ([3] and personal observations). Another important feature of IL-12Rβ1 deficiency is that it is an autosomal recessive disorder, requiring both alleles to be affected in order to have an effect. Heterozygous parents and siblings with the same mutations do not have an immunological defect or clinical symptoms.

In the child described in this report no mycobacterial or salmonellae infections, typical for IL-12Rβ1 deficiency, were found. In addition, the infection was confined to the lung, a tissue normally not affected in these patients. The high IgE levels that were present in the child and the family history of allergic disorders are not a feature seen in IL-12Rβ1 deficiency.

No functional immunological analyses to determine whether the IL-12/IFN-γ pathway was affected were performed in the child [1]. The only experiment performed that, at best, provided circumstantial evidence that the *IL12RB1* gene might be affected is the absence of an *IL12RB2* transcript in PBMCs from the patient stimulated, not with a relevant stimulus such as IL-12 or IL-23, but with a mitogen. Subsequent sequencing of the *IL12RB1* transcript revealed a heterozygous variation, 531G>A, which leads to the amino acid substitution R156H. The variation was not found in 100 chromosomes of unrelated individuals, after which it was concluded that this must be a mutation [1].

The R156H variation in IL-12Rβ1 has been reported before and is known to be present in 11.3% of North Americans (4550 chromosomes analysed) and 15% of Europeans (1285 chromosomes analysed) [4]. An aspect that may have confused the authors is that the position of variations is, as a rule, counted from the first A of the ATG translation initiation codon, thus designating the variation found in this child as 467G>A in stead of 531G>A [5]. We have analyzed R156H, as well as several other amino acid variations in IL-12Rβ1, in a retroviral expression system and found the R156H variation to be expressed on the cell membrane and not to have any effect on IL-12 responses as determined by measuring IFN-γ and IL-10 production, thus concluding R156H is most likely a harmless polymorphism [6].

Regardless of the literature provided here, one can analyze novel variations with the prediction programs SIFT (sift.jcvi.org/) and Polyphen (genetics.bwh.harvard.edu/pph2/) to determine whether the substitution is more likely to be a mutation or a polymorphism. According to SIFT, which predicts the effect of amino acid substitutions on the basis of evolutionary conservation, the R156H variation is tolerated. According to Polyphen, predicting the possible impact of an amino acid substitution on the structure and function of a human protein using straightforward physical and comptive considerations, the R156H variation is benign. In order to, in the future, facilitate finding out whether a variation is already known to be a mutation, a polymorphism or a variation with an unknown effect, a database specific for *IL12RB1* variations was recently launched at: http://www.LOVD.nl/IL12RB1.

In conclusion, we feel there is no reason to suspect a defect in IL-12 signaling based on the clinical data, nor evidence for a functional defect in IL-12 signaling in this patient. In addition, the variation identified is, based on various data sources, understood to be a polymorphism. Without any functional evidence that R156H is a mutation, the current claim is not substantiated.

Reply - Loredana Palamaro, Giuliana Giardino, Francesca Santamaria, Rosa Romano, Anna Fusco, Silvia Montella, Mariacarolina Salerno, Matilde Valeria Ursini and Claudio Pignata

Dear Editor,

In their comment to our case report on a child with interleukin 12 receptor alteration, van de Vosse and van Dissel raised the important issue of defining the causative relationship between a genetic variation and the phenotypic expression of a clinical entity, stating that the R156H in IL-12 beta1 receptor chain, reported in the patient described, is not a mutation but a variation also present in otherwise healthy individuals.

In principle, we agree with the Authors, in that to define the R156H allele as causative of the disease may be considered an overstatement. However, it should be emphasized that from recent whole genome sequencing studies, the analysis of the association between genetic variants and complex traits is establishing as a powerful strategy to assign a role also to variations otherwise considered polymorphisms. There are additional examples of genetic alterations, referred as polymorphisms and, therefore, with a prevalence in the general population higher than 1%, which are now considered important genetic susceptibility factors. The Ala91Val variation of the perforin gene is present in healthy controls, but is considered an important genetic element to confer susceptibility to lymphohistiocytosis [7-9].

In our case report we used the potent mitogen stimulation, which is widely recognized as a trigger of a Th1 polarization, to document the absence of the expression of the high affinity IL-12 R, consisting of both beta 1 and beta 2 chains. Certainly, a functional evidence of impaired IL-12 signaling could help to clarify the relationship between the genetic variation and the clinical phenotype. Unfortunately, this information is not available at moment.

As for the molecular analysis, this was performed on cDNA starting to count from the +1 site. However, this different enumeration does not interfere with the aminoacid substitution, which remains the same as before (R156H). The important finding of van de Vosse that this substitution doesn’t affect IL-12 R signaling would suggest that this variation in our case exerts a summatory effect, as a genetic cofactor, along with an additional and still unidentified molecular alteration of the same pathway.

## Competing interests

The authors declare that they have no competing interests.
